# Single-shot in situ readout of a spin qubit unit cell

**DOI:** 10.1038/s41928-026-01654-9

**Published:** 2026-06-17

**Authors:** Pierre Hamonic, Mathieu Toubeix, Guillermo Haas, Jayshankar Nath, Matthieu C. Dartiailh, Biel Martinez, Benoit Bertrand, Heimanu Niebojewski, Maud Vinet, Christopher Bäuerle, Franck Balestro, Tristan Meunier, Matias Urdampilleta

**Affiliations:** 1 Institut Néel, Université Grenoble Alpes, CNRS, Grenoble INP, Grenoble, France; 2 CEA-List, Université Grenoble Alpes, Grenoble, France; 3 Quobly, Grenoble, France; 4 CEA-Leti, Université Grenoble Alpes, Grenoble, France

**Keywords:** Quantum information, Electronic devices

## Abstract

Spin qubits based on semiconductor quantum dots can offer high coherence times and gate fidelities, and are a potential platform for quantum computation. However, scaling up the number of qubits to the level required for fault-tolerant quantum computing is challenging and will require single-shot, low-footprint qubit readout. Here we report single-shot readout of a spin qubit unit cell using in situ dispersive readout. Our compact, foundry-fabricated unit cell is composed of two electron spins with a controllable exchange interaction, and with it we demonstrate initialization, single-shot readout and a two-electron entangling gate. From exchange oscillations, we extract a spin visibility of 80.7(8)% in the coherent control of a qubit, despite a relatively small lever arm to the resonator gate (less than 0.15(5) eV V^−1^). We show that the unit cell can be operated at up to 1 K with low charge noise levels, extracted using free induction decay.

## Abstract (web summary)

The state of a foundry-fabricated spin qubit unit cell, which is composed of two electron spins with a controllable exchange interaction, can be read out in a single shot using gate reflectometry.

## Main

Silicon spin qubits offer strong potential for scalability due to their compatibility with complementary metal–oxide–semiconductor (CMOS) processes^1–7^. The use of industrial manufacturing techniques could also lead to improvements in device reliability and quality^5,8–12^. Recent work has, for instance, demonstrated high-quality two-qubit gates in 300-mm foundry-fabricated unit cells^13^. However, the need for charge-sensor-based qubit readout systems, which require additional electrostatic tuning, hinders scaling at the unit-cell level^14^. In situ qubit readout using gate-based dispersive methods could provide a scalable alternative^15–17^, particularly if the high electrostatic coupling of industrial gate stacks is exploited^18–21^. However, single-shot gate-based readout of a qubit remains a challenge.

Single-shot readout provides shot-by-shot state discrimination^22^. It can also enable mid-circuit measurements^23^ and real-time feedback protocols^24^. In addition, it is essential for error-correction schemes relying on fast, repeated ancilla measurements^25^. Gate-based dispersive sensing of spin qubits has been developed^26–28^. However, single-shot, non-destructive readout of a coherent qubit state using only its accumulation gate remains restricted by several fundamental and technical challenges. First, constructing a resonator with a sufficiently large dispersive signal while remaining far from the charge transition is difficult, as operating at frequencies close to the tunnel-coupling-induced energy splitting can lead to readout-induced transitions^27^. Second, demonstrations of single-shot spin readout often rely on large tunnel couplings^26,28^, which are incompatible with the moderate exchange interactions required for two-qubit gate operations. Finally, current dispersive readout schemes are limited to the singlet and triplet minus subspace {S, T_−_}, leaving the unpolarized triplet state T_0_ inaccessible, which limits complete two-qubit state characterization.

In this Article, we report a resonator optimized to maximize the dispersive shift while preserving adiabaticity. With this system, we demonstrate single-shot discrimination of the full singlet–triplet states {S, T_±_, T_0_} in a regime compatible with two-qubit interactions, enabled by precise control of the tunnel coupling *t*_c_ via the confinement potential *C*. Our approach does not need an external charge sensor and provides a compact qubit unit cell with a minimal number of electrostatic controls. We fit coherent oscillations and extract a coherence time of 423(5) ns for an exchange value of 68(1) MHz, which corresponds to a quality factor *Q* ≈ 28. The qubit exhibits good performance metrics and maintains reasonable visibility up to 1 K, and could be suitable for cointegration with dissipative cryoelectronics^29–31^.

## The spin qubit unit cell

**Fig. 1:**
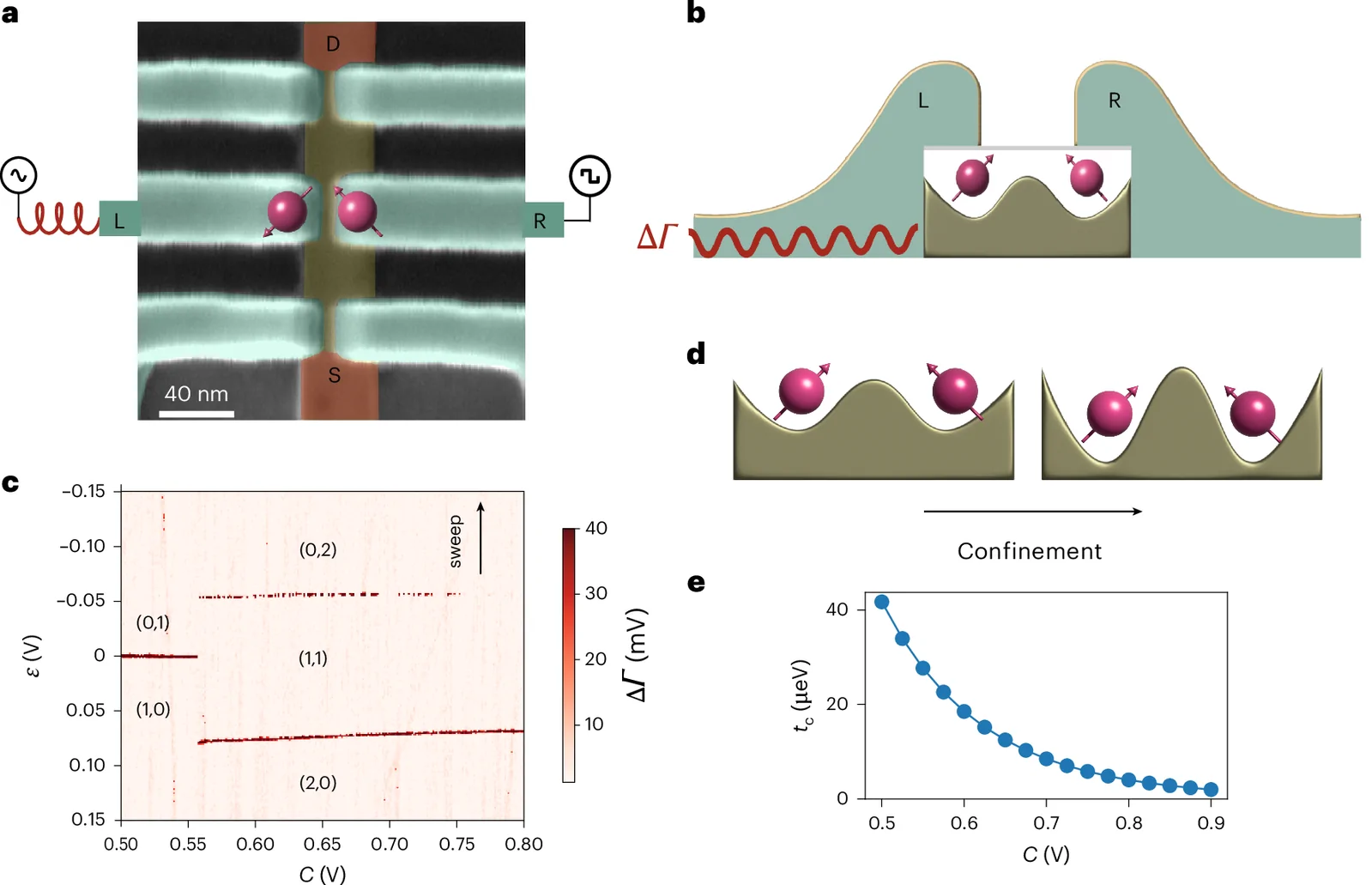
The foundry-fabricated spin qubit unit cell. **a**, Scanning electron microscopy of the split-gate device with false colours. The silicon channel (khaki) is covered by split gates (pastel green) with a SiO_2_/TiN/polysilicon gate stack. A double quantum dot is formed under gates L and R with a total of two electrons loaded from the reservoirs (S and D). The L gate is connected to a tank circuit formed by an inductance (depicted in red) and the device’s parasitic capacitance to ground. The R gate is connected to a bias tee to allow application of rapid pulses to electrically manipulate the singlet–triplet qubit. **b**, Artistic representation of a cross-section of the device showing the isolated double dot formed between the two split gates (in green) and the location of the two quantum dots (represented by the electron spin). **c**, Charge stability diagram obtained by measuring Δ*Γ* on the resonator as *ε* is swept and *C* is varied. The ICTs delimiting the three charge regions are clearly visible for two electrons. The diagram also shows a single ICT at shallow confinement (below *C* = 0.55 V) where one of the two electrons has escaped the double quantum dot. **d**, Artistic representation of how confinement potential affects the height of the tunnel barrier between the two quantum dots. **e**, Simulated dependence of *t*_c_ on *C*. Simulation details are given in Supplementary Section 4 and Supplementary Fig. 4.

We used a device patterned on a 300-mm wafer in the CEA-Leti industrial research foundry using fully depleted silicon on insulator technology (Fig. 1a)^32^. It features a 40-nm-wide 10-nm-thick silicon channel (Fig. 1b). The two extremities of the channel, labelled source (S) and drain (D), are n doped to create reservoirs of electrons; they are separated from the substrate by a 145-nm layer of buried oxide. Titanium nitride and polysilicon gates are deposited on top of the channel, over a 6-nm isolation layer of thermally grown dioxide. A combination of deep-ultraviolet and electron-beam lithography is used to pattern the gate structure, as described in refs. ^33,34^.

All gates are 40 nm wide and spaced 40 nm apart in both the longitudinal and transverse directions. To prevent unwanted doping of the channel, silicon nitride spacers (35 nm thick) are deposited between the gates before doping the reservoirs. Finally, the whole device is encapsulated and the gates are connected to aluminium bond pads through standard Cu-damascene back-end-of-line processing.

The unit cell used here contains two gates labelled L and R for left and right, in reference to their positions with respect to the silicon channel (Fig. 1a,b)^35^. L is connected to a superconductive inductor with *L* = 69 nH; together with the parasitic capacitance *C*_p_ = 0.25 pF, it forms a resonator with resonance frequency *f* = 1,148 MHz and quality factor around 50. The inductor is further connected to a bias tee with cutoff frequency *f*_c_ = 100 kHz to allow application of both a reflectometry tone and a direct-current voltage. The amplitude variation (Δ*Γ* in Fig. 1b,c) of the reflected radio-frequency signal close to the resonance frequency is determined using analogue demodulation at room temperature. R is connected to a bias tee with the same cutoff frequency, this time to allow fast manipulation pulses to be applied together with a direct-current voltage. The other gates are direct-current biased and only relevant during the loading sequence and for isolation of the unit cell.

A key advance of the present device architecture is the ability to fully isolate the double quantum dot from the source and drain reservoirs using additional lateral gates. This regime allows deterministic tuning of the exchange interaction even in the absence of a dedicated tunnel barrier, providing a level of electrostatic control that was not accessible in the previous generation of devices^21,36^. In particular, we can load a finite number of electrons into L and R and then isolate a double quantum dot from the reservoirs following our published procedure^32^. Hereafter, we work with two electrons in the isolated double quantum dot^37^; there are thus three possible charge states in the isolated regime: (0,2), (1,1) and (2,0). To tune the double quantum dot, we apply the voltages *V*_L_ and *V*_R_ on left and right gates respectively and we define the detuning $\varepsilon =\frac{{V}_{{\rm{L}}}-{V}_{{\rm{R}}}}{2}$ and the confinement potential $C=\frac{{V}_{{\rm{L}}}+{V}_{{\rm{R}}}}{2}$, as the two gates have similar lever arm ratios. Figure 1c shows the resonator response as *ε* and *C* vary. A few parallel interdot charge transitions (ICTs) are observed, separating the possible charge states. When two electrons occupy the double quantum dot, two ICTs are visible, separating the (0,2), (1,1) and (2,0) charge configurations. These ICTs tend to disappear toward high potential depth (large *C* voltages). We interpret this effect as a result of a drop-off in tunnel coupling, to the point that the transitions are no longer detectable. Indeed, a large potential depth corresponds to strong confinement, which creates a larger barrier between the dots and thus necessarily reduces tunnel coupling (Fig. 1d)^38^. This is further confirmed by simulations presented in Fig. 1e, where the evolution of tunnel coupling is calculated as a function of the confinement voltage (Supplementary Section 4 and Supplementary Fig. 4). We exploit this confinement to fine-tune the tunnel coupling and the exchange interaction between two electrons in our device. On the other hand, when the confinement becomes too shallow (lower *C* voltages), the chemical potential rises above the Fermi sea, and one of the two electrons leaves the double quantum dot. As a consequence, below *C* = 0.55 V only the (0,1) and (1,0) charge configurations are visible.

## Single-shot readout and initialization

We read our qubit by measuring the difference in reflection of a radio-frequency tone on the tank circuit at a frequency close to the resonance frequency *f* = 1,148 MHz. The resonator is operated in the dispersive regime, where the probe frequency is well below the tunnel coupling in the standard spin qubit operating regime (*t*_c_ < 20 μeV, corresponding to 5 GHz), ensuring that the readout is sensitive only to the dispersive response. In this regime, a small excitation is used to probe the dispersion relation of the quantum states. Therefore, when the detuning energy approaches an ICT, an additional quantum capacitance *C*_Q_ arises due to the tunnel coupling^39^. This alters the resonance frequency of the resonator and, consequently, the reflected signal. In our device, this shift, Δ*Γ*, is measured through homodyne detection.

This detection scheme enables spin readout of the unit cell as follows: due to Pauli spin blockade (PSB), only singlet states can readily tunnel from (1,1) to (0,2) or (2,0) and give finite quantum capacitance. In contrast, triplet states are blocked, and thus present zero quantum capacitance, leaving the radio resonator signal unchanged. Singlet and triplet states can therefore be readily distinguished at the ICT.

**Fig. 2:**
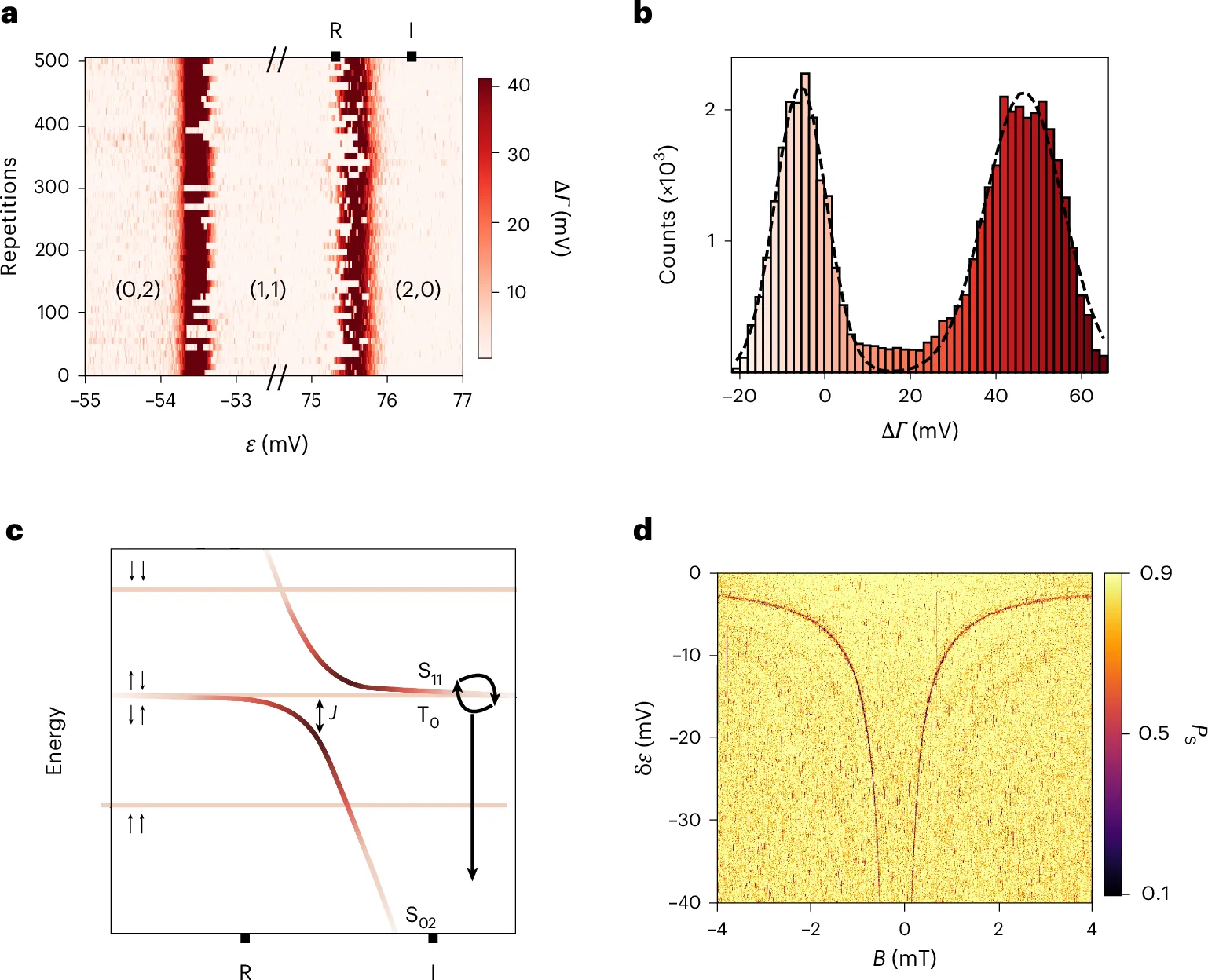
Single-shot readout and initialization. **a**, Horizontal cross-section of Fig. 1c taken at *C* = 0.7 V for 500 repetitions. On both ICTs, (0,2) ↔ (1,1) and (1,1) ↔ (2,0), thermal singlet to triplet excitations combine with PSB to produce pixels with zero signal. **b**, Histogram obtained by binning the repeated signal at measurement position R on the (1,1) ↔ (2,0) transition to probe the singlet and triplet populations. The dashed line corresponds to a fit to the probability distribution (Supplementary Section 4 and Supplementary Fig. 4). **c**, Energy diagram for the qubit, highlighting the relevant states (the singlets S_11_ and S_02_ and the unpolarized triplet T_0_, and the two polarized triplets ↑↑ and ↓↓, also labelled T_−_ and T_+_ hereafter) and the positions for initialization (I) and readout (R). **d**, Spin-funnel experiment showing the singlet return probability *P*_s_ as a function of magnetic field *B* and detuning variation δ*ε*. The two lines show the position of the S–T_−_ anticrossings. From their splitting at zero field and large detuning, we extract the residual exchange interaction at zero detuning (deep in (1,1)), *J*_0_ = 60 MHz. The field is slightly offset due to hysteresis in the magnetic coil.

PSB manifests as stochastic signal jumps in the ICT diagram. As detuning is scanned across the ICTs (1,1) ↔ (0,2) and (1,1) ↔ (2,0) (Fig. 2a), the finite and zero quantum capacitance are represented as red and blank pixels, respectively. We attribute the fluctuations observed to thermally excited triplet states that relax at a rate comparable to the measurement time (1 ms per pixel).

The PSB signature is the same for both transitions in Fig. 2a. Consequently, although in the rest of this Article results for the (1,1) ↔ (2,0) ICT are presented, the reader should note that similar results were obtained for the (0,2) ↔ (1,1) transition (Supplementary Section 2 and Supplementary Fig. 2).

To characterize the single-shot readout fidelity, we exploit PSB at the ICT. We place our measurement position at the point of maximum contrast on the (1,1) ↔ (2,0) transition and record data for 10,000 samples. The data are illustrated in the histogram displayed in Fig. 2b. Fitting the histogram with two bell curves and adjusting the threshold to maximize visibility, we obtain a charge fidelity of 97(1)% for an integration time of 100 μs. We use the same method hereafter to define the threshold and extract single-shot measurements (see Supplementary Section 3 and Supplementary Fig. 3 for the detailed analysis).

We next apply the readout method as part of a spin-funnel experiment^40^. We start by initializing a singlet state by spending time (*t* = 100 μs) at a relaxation hotspot in (2,0), labelled I in Fig. 2a,c, until all triplets have relaxed. At low field, the triplet relaxation rate is strongly enhanced by spin-state mixing^41^ due to spin–orbit coupling^42^, or residual hyperfine interactions. By monitoring these parameters, we determine a greater-than-95% initialization fidelity in the singlet state. However, this fidelity degrades as the magnetic field increases, due to leakage to the T_−_ state. We then pulse various detunings in (1,1), finally coming back to the readout position, labelled R in Fig. 2a,c. When the pulse amplitude matches the detuning position of the S–T_−_ anticrossing, the singlet rapidly mixes with the triplet, and we observe a smaller return singlet probability. By varying the magnetic field, and therefore the position of the S-T_−_ anticrossing, we can trace the singlet energy branch. Figure 2d presents the results of one such experiment, where the singlet return probability is plotted as a function of both magnetic field and detuning pulse amplitude for a square pulse duration of 400 ns. This measurement allows us to extract the residual exchange interaction in (1,1), *J*_0_ = 60 MHz for *C* = 0.7 V. However, this value can be tuned by adjusting the confinement potential despite the absence of a coupling gate.

## Spin visibility of coherent exchange oscillations

Next, we implement a qubit on the natural basis formed by the singlet (S) and unpolarized triplet (T_0_) states of our double quantum dot (see Fig. 2c)^43^. We then operate this qubit in the two-spin subspace using only gate voltages, and use gate reflectometry to read it.

**Fig. 3:**
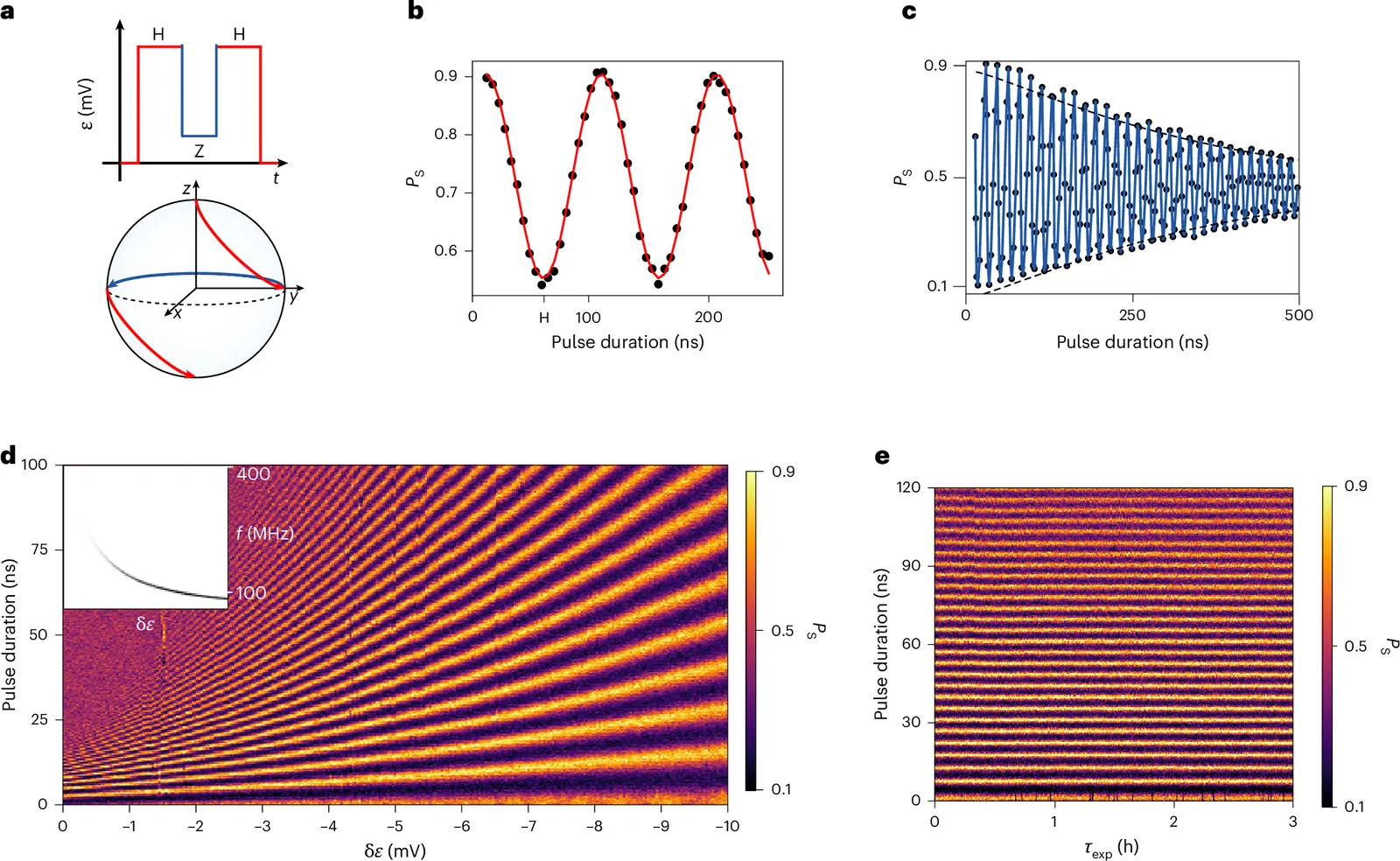
Spin visibility of exchange-driven coherent oscillations at low temperature. **a**, Pulse sequence used to measure exchange oscillation. A Hadamard gate, H, is first applied to bring the state to the equatorial plane. The detuning is then modified to apply a phase gate, Z. The qubit is subsequently allowed to evolve in the equatorial plane before it is projected back to the *z* axis by applying a second H gate. A specific case of an X = HZH gate is presented on the Bloch sphere. **b**, Oscillations of *P*_s_ in the case where *J* matches Δ*B*_z_. The black dots are experimental points and the red line is a fit to the coherent evolution using a cosine function. This curve was used to calibrate the H gate. **c**, Exchange oscillations for *J* = 68(1) MHz reveal a spin visibility of 80.7(8)%. The dashed envelope corresponds to a fit with exp(−*τ*/*T*_2_*)^α^, with *α* = 1.3 and the decoherence time *T*_2_* = 419(5) ns. The extracted quality factor *Q* = 28. The black dots are experimental points and the blue line is a guide for the eye. The dashed line is fit to the coherent evolution using a Gaussian decay. **d**, Exchange oscillations obtained using the pulse sequence described in **a**. Inset: Fourier transform of the exchange oscillations versus detuning, which gives the evolution of the frequency of exchange oscillations, *f*. **e**, Exchange oscillations recorded at *J* = 220(3) MHz as a function of the total experimental time, *τ*_exp_, for a total of 3 h.

Despite the absence of a coupling gate, we can tune the exchange interaction over one order of magnitude by adjusting the confinement potential. We tune the residual exchange interaction in (1,1) around *J*_0_ = 6 MHz at *C* = 0.8 V; we can produce exchange oscillations in a Ramsey-like experiment with the pulse sequence described at the top of Fig. 3a. As before, we start by initializing a singlet state in (2,0) using the method described for the funnel experiment (point I in Fig. 2c). We then create a superposition of states by executing a Hadamard gate (H), see Fig. 3b. The H gate is implemented by pulsing to a position where the exchange energy *J* is equal to Δ*B*_z_, the difference in Zeeman energy between the two dots, *J* = Δ*B*_z_ = 6 MHz at 1 T, and staying there for a time ${\tau }_{{\rm{H}}}=1/2\sqrt{{J}^{2}+\Delta {B}_{{\rm{z}}}^{2}}$ (Fig. 3b). During this time, the initial singlet rotates by π around an axis **y** + **z** to end up in the |↑↓〉 state^44,45^. Applying a Z phase gate changes the phase of the qubit by pulsing to a detuning where *J* ≫ Δ*B*_z_. We then let the system freely evolve for a time *τ*_p_, during which it accumulates a phase at a rate proportional to $\sqrt{{J}^{2}+\Delta {B}_{{\rm{z}}}^{2}}\approx J$. Applying another H gate at the end of this free evolution returns the system to the S–T_0_ basis, allowing its measurement during the readout step (R in Fig. 2c). The singlet probability determined with respect to the duration of the free evolution step is presented in Fig. 3c. By changing the detuning at which the Z gate is performed, we can change the frequency of the exchange oscillations (Fig. 3d), producing oscillations at rates of up to 300 MHz.

On this coherent oscillation, spin visibility is around 80.7(8)% for an integration time of 100 μs. This is in agreement with a spin readout fidelity of around 91(2)%, which is less than the 95% charge readout fidelity previously obtained (Fig. 2b). This degradation may be linked to the reduction in signal due to the relatively high magnetic field used in the experiment (1 T). In effect, the magnetic field causes a shift and a reduction in the quality factor of the resonator (Supplementary Section 8 and Supplementary Fig. 8), halving the signal amplitude.

We next characterize the qubit’s quality in terms of decoherence induced by charge noise at low temperature. By fitting the coherent oscillations in Fig. 3 (ref. ^46^), we obtain a coherence time of 423(5) ns for an exchange value of 68(1) MHz. These values give a quality factor of around *Q* = 28, which is among the best reported values in MOS devices for exchange oscillations operated with detuning control^47^. However, control of the exchange interaction at the symmetric operation point by pulsing the tunnel barrier leads to higher fidelity due to reduced sensitivity to charge noise.

While our current set-up successfully demonstrates coherent qubit operations and single-shot readout, the implementation of randomized benchmarking on the singlet–triplet qubit is limited by the instrumentation and control architecture rather than intrinsic qubit properties, as discussed in Supplementary Section 6 and Supplementary Fig. 6.

However, from the extracted exchange oscillation *Q* factor, we can roughly estimate the fidelity of a maximally entangled gate comprising two Loss–DiVincenzo qubits such as the $\sqrt{{\rm{SWAP}}}$ gate. Using *F* = 1 − *t*_g_/*T*_2_*, where *t*_g_ is the gate time^47^, fidelity of the order of 99% can therefore be expected. Full characterization of the fidelity of the two-qubit gate would require randomized benchmarking combined with arbitrary initialization of both spins. Nevertheless, we take this first estimation of fidelity as a demonstration of the high quality of the device in terms of charge fluctuations.

To investigate the charge noise for the qubit unit cell more accurately, we probe electrical fluctuations using exchange oscillations^44,45,48^. We start by characterizing the qubit’s sensitivity to noise by exploiting the dependence of the exchange energy on detuning. By performing a Fourier transform of the oscillations shown in Fig. 3c, we extract the value of the detuning-dependent exchange coupling *J*, which follows the expected trend $J=\varepsilon +\sqrt{{\varepsilon }^{2}+4{t}_{{\rm{c}}}^{2}}$ (Supplementary Section 4 and Supplementary Fig. 4). Using a sensitivity calibration curve built from the dependence of *J* on detuning, sensitivity values of up to d*J*/d*ε* = 1 MHz μeV^−1^ are reached. Figure 3e presents exchange oscillations recorded over 3 h at points of high sensitivity (1 MHz μeV^−1^). The plot reveals extremely stable oscillations, confirming the good performance of the device at 80 mK.

## Hot qubit operation

1-K operation of spin qubits markedly relaxes experimental constraints^3,49^. In particular, it makes it possible to work in cryogenic systems with high available cooling power, a crucial milestone on the pathway toward the cointegration of classical dissipative electronic chips and quantum chips^30,31^.

**Fig. 4:**
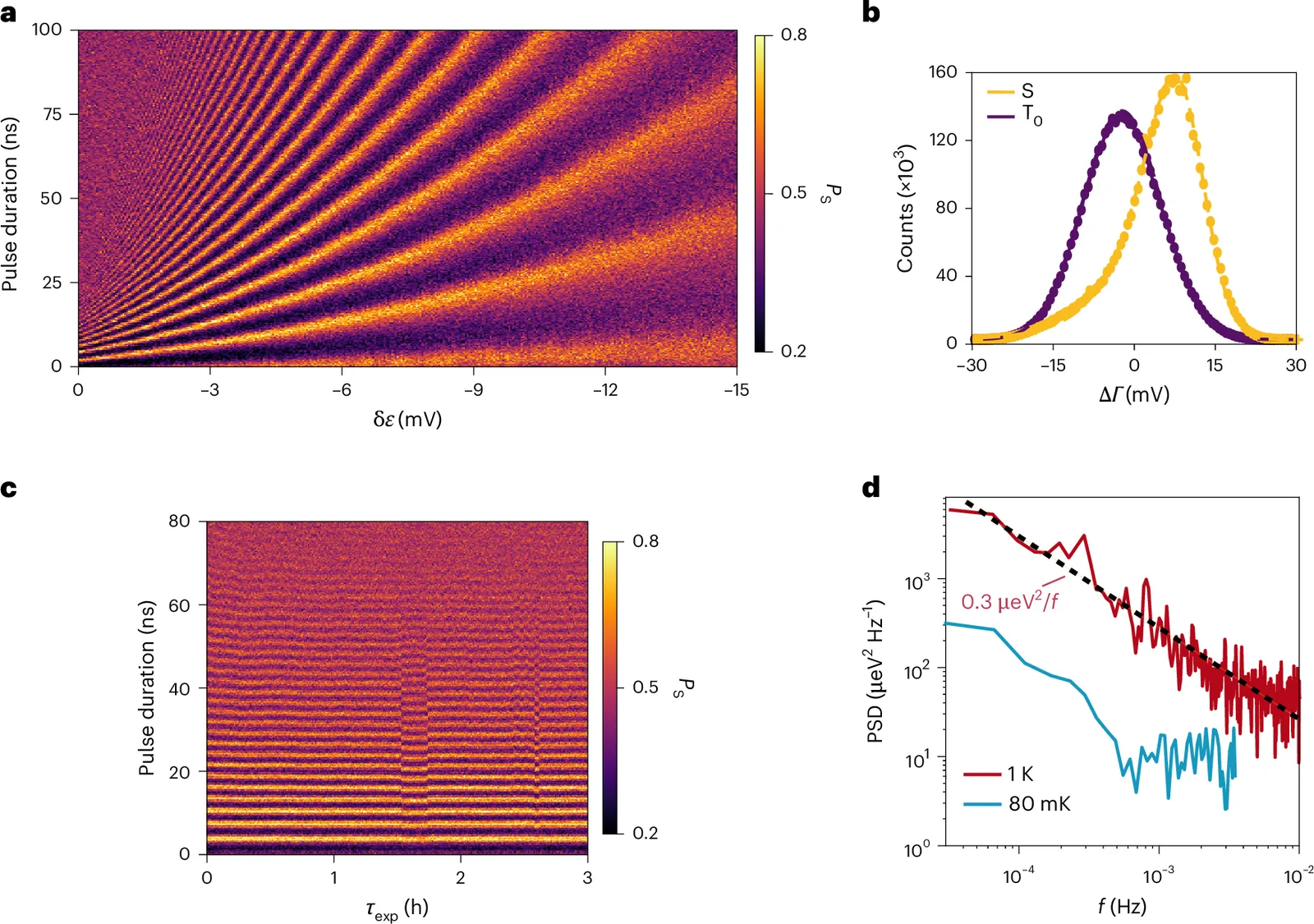
Hot operation of the qubit. **a**, Exchange oscillations at 1 K. **b**, Histograms showing the initialized singlet state (yellow) and the triplet state (purple) produced after application of an HZH to the singlet state. The triplet and singlet distributions are fitted using a simple Gaussian and a Gaussian convoluted with an exponential decay respectively to account for spin relaxation during the measurement time (Supplementary Section 7 and Supplementary Fig. 7). This gives a relaxation time *T*_1_ of 732(17) μs at the readout point. The extracted fidelity from these distributions is 72.9(4)%, in agreement with the 52(2)% visibility extracted by fitting the coherent oscillations in **a**. **c**, Exchange oscillations recorded at *J* = 310(2) MHz over 3 h. **d**, PSD of the charge noise at 1 K extracted from the free induction decay measurement presented in **c** and that obtained at 80 mK. The 1-K PSD trend follows the black dashed line, corresponding to a fit to *A*/*f*, with *A* = 0.33(17) μeV^2^ Hz^−1^. The extraction of the PSD at 80 mK is limited by the resolution of the Fourier transform above 5 × 10^−3^ Hz.

We characterize our qubit unit cell at 1 K in terms both of readout and of coherent control. Figure 4a shows the coherent exchange oscillations as a function of detuning at 1 K. Due to a reduction in readout signal amplitude relative to the measurements performed at lower temperature, a spin visibility of 52(2)% is extracted by fitting the exchange oscillations. Figure 4b shows the distributions, which are fitted using the method detailed in Supplementary Section 3 and Supplementary Fig. 3. We extract a fidelity of 72.9(4)% and a visibility of 48.4(4)% in agreement with the above measured visibility. A more detailed analysis of these distributions reveals that only the signal amplitude has changed by a factor of 6, not the noise contribution to the signal-to-noise ratio (width of the distributions), which is only limited by the noise temperature of the amplification chain (6.3 ± 1.1 K). As the temperature rises, the singlet excited state becomes thermally populated. The excited and ground singlets have opposite shift contributions, which leads to a decrease of thermal averaged dispersive shift. A numerical simulation of the amplitude of the dispersive shift as a function of both temperature and tunnel coupling is presented in Supplementary Section 5 and Supplementary Fig. 5. It shows the expected sixfold reduction of the signal strength, in agreement with our experimental observation.

To extract the charge noise for the qubit unit cell more quantitatively at 1 K and for low temperature, we exploit exchange oscillations to probe electrical fluctuations. Figure 4c shows the exchange oscillations measured at points of high sensitivity at 1 K, recorded over 3 h. From these measurements, we can extract the power spectral density (PSD) (Fig. 4d), which is plotted in comparison with that extracted at 80 mK.

To do so, we first apply a Fourier transform to the time-dependent variation of the exchange energy, and normalize the result using a sensitivity to detuning noise of 1 MHz μeV^−1^ (extracted from the derivative of *J*(*ε*) in Supplementary Fig. 4). However, this method has a limit on sensitivity given by the finite length of the exchange oscillations being recorded and Fourier transformed. This can be seen on the low-temperature PSD, which saturates at 10 μeV^2^ Hz^−1^ above 5 mHz. However, on the 1-K PSD we can still observe a 1/*f* trend that confirms the electrical origin of the noise for large exchange interactions. Moreover, the amplitude of charge noise detuning extrapolated at 1 Hz is 0.33(17) μeV^2^ Hz^−1^ at 1 K. This value is similar to that obtained at lower temperatures with other semiconductor spin qubit platforms^45,50,51^, and is two orders of magnitude smaller compared with the previous generation of devices. This improvement is attributed to two different factors. The first is the changes in the materials stack and reservoir definition. In contrast to refs. ^21,36^, which employed a mixed SiO_2_/HfO_2_ gate dielectric, the current devices rely exclusively on SiO_2_, leading to a large reduction in charge fluctuations and enhanced long-term stability. Second, we believe that the improvement is also due to the isolation from reservoirs; charge noise is reduced by decoupling from two-level systems located at the edges of the source and drain junctions^32^. Although a detailed study of the origin of charge noise is beyond the scope of this work, these modifications lead to a clear suppression of charge noise, enabling stable operation in the few-electron regime.

## Conclusion

We have reported the single-shot measurement of a spin qubit using in situ gate reflectometry. By fitting the envelope of the oscillations, we extract a visibility of 80.7(8)% in the coherent control of a qubit, despite its relatively small lever arm to the resonator gate (<0.15(5) eV V^−1^). Using this readout approach, we show that an isolated double quantum dot in a foundry-fabricated device can form a qubit unit cell operating effectively at temperatures up to 1 K. This cell is compatible with both two-electron spin coherent control and in situ readout. The coherent control exhibits good metrics in terms of quality factor, coherence time and robustness to charge noise both at low temperature and at 1 K.

The operation of spin qubits at elevated temperatures, such as 1 K, is a reasonable trade-off between experimental practicality and qubit performance^3^. While higher temperatures simplify the integration with classical cryoelectronics^31^ and reduce cooling requirements, they also introduce challenges for qubit coherence and readout fidelity. In our experiments, raising the temperature from 80 mK to 1 K primarily affects the resonator response and qubit dynamics rather than the noise floor of the readout chain. The effective noise temperature of the radio-frequency amplification chain remains dominated by the 4-K high-electron-mobility transistor amplifier, meaning that the additional noise at 1 K is negligible. However, the dispersive signal amplitude decreases due to the thermal population of the excited singlet state, which reduces the overall readout fidelity. While a sixfold reduction in signal strength is observed when operating at 1 K, the coherence time only decreases by approximately a factor of 2. This is consistent with a 1/*f* noise, which leads to 1/*T*_2_* ∝ √*S*, where *S* is the PSD^52^. Assuming that the 1/*f* noise evolves linearly with temperature, this results in 1/*T*_2_* ∝ √*T*, consistent with experimental observations. Therefore, to benefit from relatively stable qubit metrics at elevated temperature, the readout fidelity must be improved. Enhancing the signal-to-noise ratio of gate-based dispersive readout is thus essential.

Such improvements can be achieved through resonator design^53^. Increasing the quality factor and characteristic impedance^27^, as well as improving stability under magnetic fields^54^, can directly amplify the dispersive shift. Further improvements may arise from material innovations—for example, the use of high-kinetic-inductance superconductors such as TiN^55^. These materials enable the fabrication of compact, CMOS-compatible resonators with footprints as small as a few square micrometres, reducing parasitic capacitances and boosting the signal-to-noise ratio^56^. Combining these strategies can lead to improved single-qubit readout and more efficient integration of large-scale qubit arrays at elevated temperature.

Beyond individual qubit performance, large-scale architectures must address both qubit coupling and readout scalability. Two main strategies emerge for our platform. The first involves operating the qubit array in a sparse mode, where neighbouring double quantum dots are left empty during two-qubit gate operations to prevent spurious electron coupling when the confinement control is pulsed^38^. This approach also enables control of the tunnel coupling without dedicated tunnel gates by tuning the confinement potential, thereby reducing the number of control parameters per qubit. The second strategy incorporates dedicated tunnel barriers between double quantum dots, allowing selective coupling while maintaining isolation. This approach is well aligned with foundry-fabricated devices, as it leverages CMOS-compatible processes to create robust and scalable qubit arrays^57^.

For readout scalability, gate-based dispersive sensing can be extended using three-dimensional integrated inductors and advanced multiplexing techniques^17^. By employing multilayer inductors and high-kinetic-inductance materials, the resonator footprint can be reduced, enabling dense qubit integration. In parallel, cryoelectronic multiplexing, where a single inductor interrogates multiple qubits through time- or frequency-domain multiplexing^11^, provides a scalable approach to maintaining high readout fidelity while minimizing hardware overhead. Together, these advances demonstrate that gate-based dispersive readout is compatible with large-scale integration and capable of supporting practical quantum processors operating at temperatures up to 1 K.

In future iterations, the qubit visibility could be increased by reducing the measurement integration time to compensate for the short relaxation time. The signal-to-noise ratio could be further improved by increasing both the quality factor and the characteristic impedance of the resonator, as well as its stability under applied magnetic fields. Finally, enabling rapid control of the tunnel coupling could help optimize the readout configuration during measurement sequences. For large-scale spin-based quantum processors, our unit cell, which does not include single-qubit control elements such as micromagnets or electron spin resonance lines, could be combined with global magnetic microwave control^58^ to achieve universal operations.

## Methods

Experiments were performed on the CMOS device shown in Fig. 1a using a dilution refrigerator with a base temperature of 30 mK. Gate-based reflectometry was achieved through analogue modulation/demodulation followed by digitization using an NI analogue-to-digital converter board. The per-point acquisition time is 100 μs unless otherwise stated. Gate voltages were applied using digital-to-analogue converters controlled with an sbRIO-9208 field-programmable gate array board and rapid pulses controlled using a radio-frequency system-on-chip with a Xilinx ZCU111 field-programmable gate array. The magnetic field is applied through a voltage-controlled Kepco current generator.

## Supplementary information

Supplementary Information Supplementary Figs. 1–8 and Sections 1–8.

## Data Availability

The data that support the findings of this study are available from the corresponding author upon reasonable request.
