# Supplementary material for: Single-shot in situ readout of a spin qubit unit cell
Source: Nat Electron. 2026 Jun 17;9(8):868–75. doi: 10.1038/s41928-026-01654-9 (PMC13493317; doi:10.1038/s41928-026-01654-9)
Supplement: Supplementary file 1 [file 41928_2026_1654_MOESM1_ESM.pdf]

nature electronics

Supplementary information

https://doi.org/10.1038/s41928-026-01654-9

Single-shot in situ readout of a spin qubit unit cell

In the format provided by the authors and unedited

SUPPLEMENTARY INFORMATION SECTION 1 : EXPERIMENTAL SETUP

The electronics of the experimental setup are presented in fig. 1. The whole experiment is orchestrated by an FPGA (Field Programmable Gate Array) which manages the other instruments. The DC voltages used to bias the dots and the isolation gates are sent using digital to analog converters (DACs) updated by the FPGA. The isolation gates described in the main text corresponds to gates L_1, L_3, R_1 and R_3 and are only connected to DC voltages as they only serve to isolate the central double dot.

For manipulating the qubit, we need to send precise short ($\approx 10\,\mathrm{ns}$) pulses. To that end, the R_2 gate (labelled simply R in the main text) is connected to a custom room-temperature electronics based on a Xilinx RFSoC ZCU-216 platform. This is done by combining the fast pulses and the DC control voltage with a bias tee with cutoff frequency $f_c = 100\,\mathrm{kHz}$.

Conversely, for reading the qubit, we need to send a radio-frequency tone and measure its reflection. To that end, the L_2 gate (labelled simply L in the main text) is connected to an RF line and the tone is combined with the DC control voltage with an identical bias tee with cutoff frequency $f_c = 100\,\mathrm{kHz}$. Next to the device chip, a superconductive inductor with $L = 69\,\mathrm{nH}$ is located and wirebonded to L_2. As mentioned in the main text, together with the parasitic capacitance $C_p = 0.25\,\mathrm{pF}$, it forms a resonator with resonance frequency $f = 1148\,\mathrm{MHz}$ and quality factor around 50. To avoid bringing noise to the device, the generated tone Γ goes through an attenuation chain totaling -60 dB across the 4K and the 80 mK stages (see fig. 1). The reflected signal is then recovered through an amplification chain that includes both a high-electron-mobility transistor (HEMT) amplifier at the 4K stage and room temperature amplifiers (RTA).

The signal is then recovered using room temperature analog demodulation giving the I and Q quadrature which are read by Analog to Digital Converters (ADC) triggered by the FPGA . The I and Q quadratures are then processed in software to give the final amplitude variation $\Delta\Gamma$ used in the main text and in fig. 1(c) and 2.

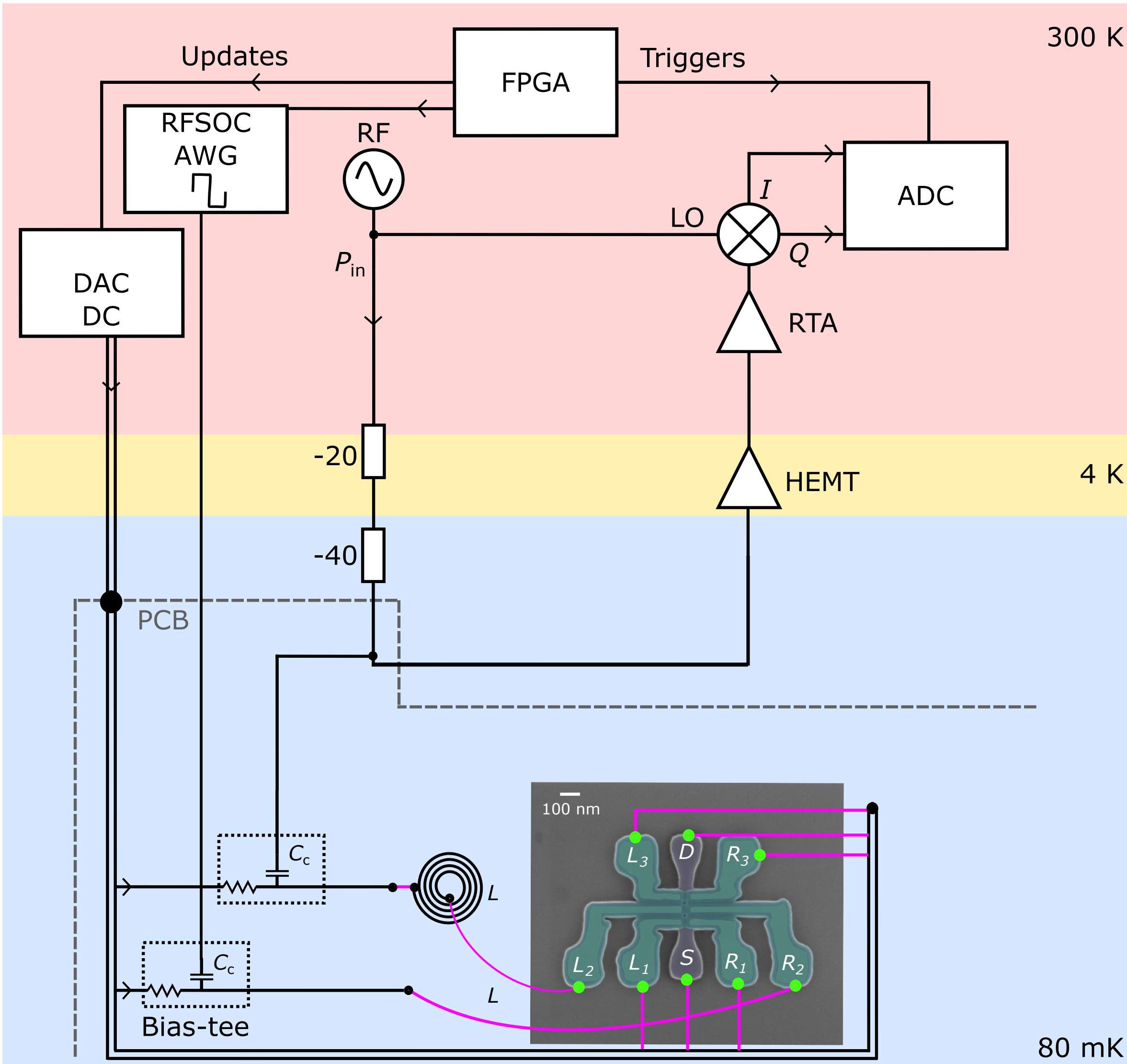

Supplementary Information Figure 1 The FPGA (Field Programmable Gate Array) controls the DAC (Digital Analog Converters) sending DC voltages to source (S), drain (D) and left and righ gates. Manipulation is achieved through fast pulses sent by the RFSoC (RF system on chip) arbitrary wave generator on R_2 through a bias tee on the printed circuit board (PCB). Readout is done by sending an RF tone on L_2 through a bias tee and a wirebonded superconductive inductance L. The local oscillator (LO) tone at power P_{in} = -30 dBm goes through -60 dB attenuation from 300K to the device and the reflected signal is amplified both at 4K with a high-electron-mobility transistor (HEMT) and 300K with room temperature amplifiers (RTA) before being demodulated and giving $\Delta\Gamma$.

SUPPLEMENTARY INFORMATION SECTION 2 : EXCHANGE OSCILLATION AT THE (0,2) TO (1,1) TRANSITION

In the main text, we have described results obtained at the optimal working point on the transition with the highest quality signal. In this section, we present the data on the second transition which is taken with a readout position at $\varepsilon = -53.5\,\mathrm{mV}$ more than 120 mV away from the working point of the main text at $\varepsilon = 75.5\,\mathrm{mV}$ (Fig. 2 (a)). By placing our measurement position at the point of maximum contrast, we record 10 000 samples. The data are illustrated in the histogram in Fig. 2 (b). We obtain at zero field a charge fidelity of 97(1)% for an integration time of 100 µs, similar to what we have for the first transition. Following the protocol described in section 3, we can make coherent exchange oscillations at 1 T by applying an Hadamard gate, letting the system freely evolve under an exchange interaction J large with respect to the difference in Zeeman energy between the two dots ΔB_z and projecting back to the S-T_0 basis. We present the data obtained in Fig. 2, showing a visibility around 52 (1)%. We can conclude that transitions between (1,1) and (0,2) or (2,0) are similar in term of tunnel coupling and exchange interaction, while these quantity are exponentially sensitive with electric field and therefore Coulomb disorder. This highlight that this foundry-fabricated devices have reached a high level of maturity in term of charge noise and disorder.

SUPPLEMENTARY INFORMATION SECTION 3 : ANALYSIS OF FIDELITY

Following the analysis of Barthel et al. [1], we fit the signal distribution of the PSB measurement using:

$$n_{\mathrm{T}}(V_{\mathrm{rf}}) = \frac{1-\langle P_{\mathrm{S}}\rangle}{\sqrt{2\pi}\sigma} e^{-\frac{(V_{\mathrm{rf}}-V_{\mathrm{rf}}^{T})^2}{2\sigma^2}}, \tag{1}$$

$$\begin{aligned} n_{\mathrm{S}}(V_{\mathrm{rf}}) &= \frac{\langle P_{\mathrm{S}}\rangle}{\sqrt{2\pi}\sigma} e^{-\frac{\tau_{\mathrm{m}}}{T_1}} e^{-\frac{(V_{\mathrm{rf}}-V_{\mathrm{rf}}^{S})^2}{2\sigma^2}} \\ &\quad + \int_{V_{\mathrm{rf}}^{T}}^{V_{\mathrm{rf}}^{S}} \frac{\tau_{\mathrm{m}}}{T_1}\frac{\langle P_{\mathrm{S}}\rangle}{\Delta V_{\mathrm{rf}}} e^{-\frac{V-V_{\mathrm{rf}}^{T}}{\Delta V_{\mathrm{rf}}}\frac{\tau_{\mathrm{m}}}{T_1}} e^{-\frac{(V_{\mathrm{rf}}-V)^2}{2\sigma^2}} \frac{dV}{\sqrt{2\pi}\sigma}, \end{aligned} \tag{2}$$

where $\langle P_{\mathrm{S}}\rangle$ is the singlet probability, V_{rf}^{S} (V_{rf}^{T}) is the signal expectation value for the non-blocked (blocked) state,$\Delta V_{\mathrm{rf}} = V_{\mathrm{rf}}^{S} - V_{\mathrm{rf}}^{T}$ is the spacing between the two expectation values,

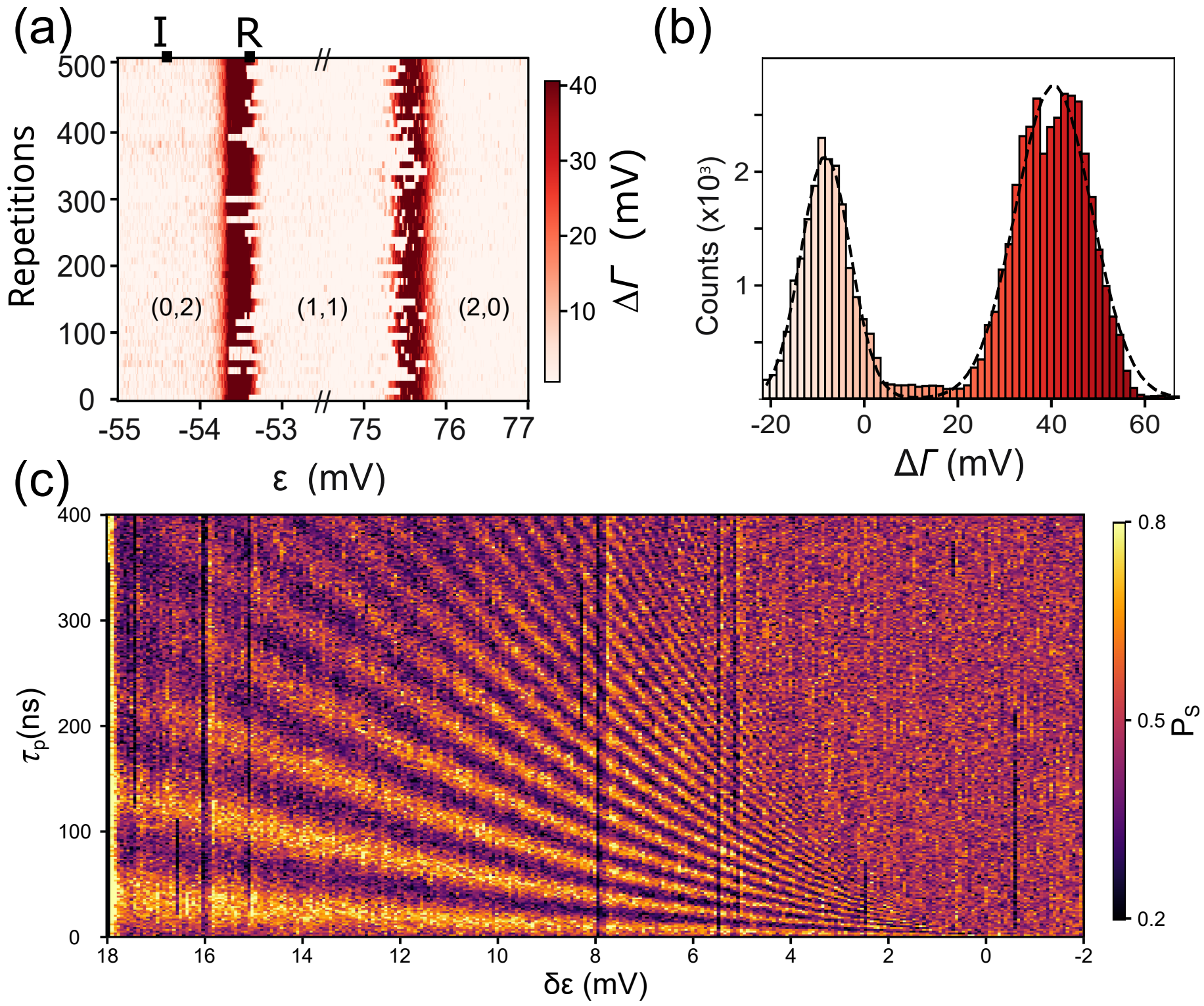

Supplementary Information Figure 2: Extended data for the (0,2)↔(1,1) transition (a) Charge stability diagram showing the new readout (R) and initialization (I) positions. **(b)** Zero-field Histogram obtained by binning the repeated signal at measurement position R on the (0,2)↔(1,1) transition to probe the singlet and triplet populations. **(c)** Coherent exchange oscillations at 1 T as a function of the detuning from the readout position for the (0,2)↔(1,1) transition.

σ is the standard deviation of the Gaussian signal distribution, $\tau_{\rm m}$ is the measurement integration time, and T_1 is the lifetime at the measurement position. While equation 1 is a Gaussian distribution, describing the blocked state signal distribution, the excited state is given by equation 2, the convolution of a Gaussian distribution with an exponential decay. We use these functions to fit the signal distribution of the data presented in the manuscript. In particular the distribution at elevated temperature which suffer from spin relaxation - while the relaxation contribution in the low temperature measurement is negligible. Note that contrary to the referenced work, our excited state is the singlet non-blocked state and the ground state is a blocked triplet state, likely the polarized T_- state. This is due to two factors : i) We work (and measure) with a much higher static magnetic field $\mathrm{B} = 1\,\mathrm{T}$ compared to $\mathrm{B} = 0.2\,\mathrm{T}$ which lowers the T_- state with respect to the polarized triplet. ii)

Our readout position, while located at the ICT, is much closer to the (1,1) region (see fig. 2 (a) and (c)) compared to the referenced work which presents measure done relatively deep in (0,2).

The definition of fidelities allows a simple metric to estimate the error of the signal assignment as blocked (non-blocked). The singlet and triplet fidelities are defined as :

$$F_{\mathrm{S}} = 1 - \int_{V_{\mathrm{T}}}^{\infty} n_{\mathrm{S}}(V) dV, \tag{3}$$

$$F_{\mathrm{T}} = 1 - \int_{-\infty}^{V_{\mathrm{T}}} n_{\mathrm{T}}(V) dV, , \tag{4}$$

where V_{T} is the threshold that separated the identification as blocked/non-blocked state. The visibility is then defined as

$$V = F_{\mathrm{S}} + F_{\mathrm{T}} - 1. \tag{5}$$

To optimize the threshold, one calculates the maximum of the visibility.

We present here a more detailed analysis of the threshold extraction at high temperature where the relaxation during measurement cannot be neglected.

SUPPLEMENTARY INFORMATION SECTION 4 : NUMERICAL SIMULATION OF EXCHANGE INTERACTION IN THE ISOLATED DOUBLE QUANTUM DOT

In this section we provide the simulated results on the dependence of exchange on detuning shown in Fig. 3 (d) of the main text. We performed numerical simulations on the realistic device geometry that we sample on a three-dimensional grid. We solved Poisson's equation within this grid to account for the realistic device electrostatics, and fed the resulting potential to an effective mass solver to obtain the single particle energies and wavefunctions. Since the experiment operates in the (1,1)-(2,0) charge configuration, correlation effects need to be accounted for. We computed the two-particle energies and wavefunctions using a full configuration-interaction (FCI) method in a constricted basis of single-particle states. We first estimated the two-particle tunnel coupling (τ_{2p}) in the experiment by fitting the dependence of J on detuning to

$$J = \frac{\delta\varepsilon}{2} + \frac{1}{2}\sqrt{\delta\varepsilon^2 + 4\tau_{2p}^2} \tag{6}$$

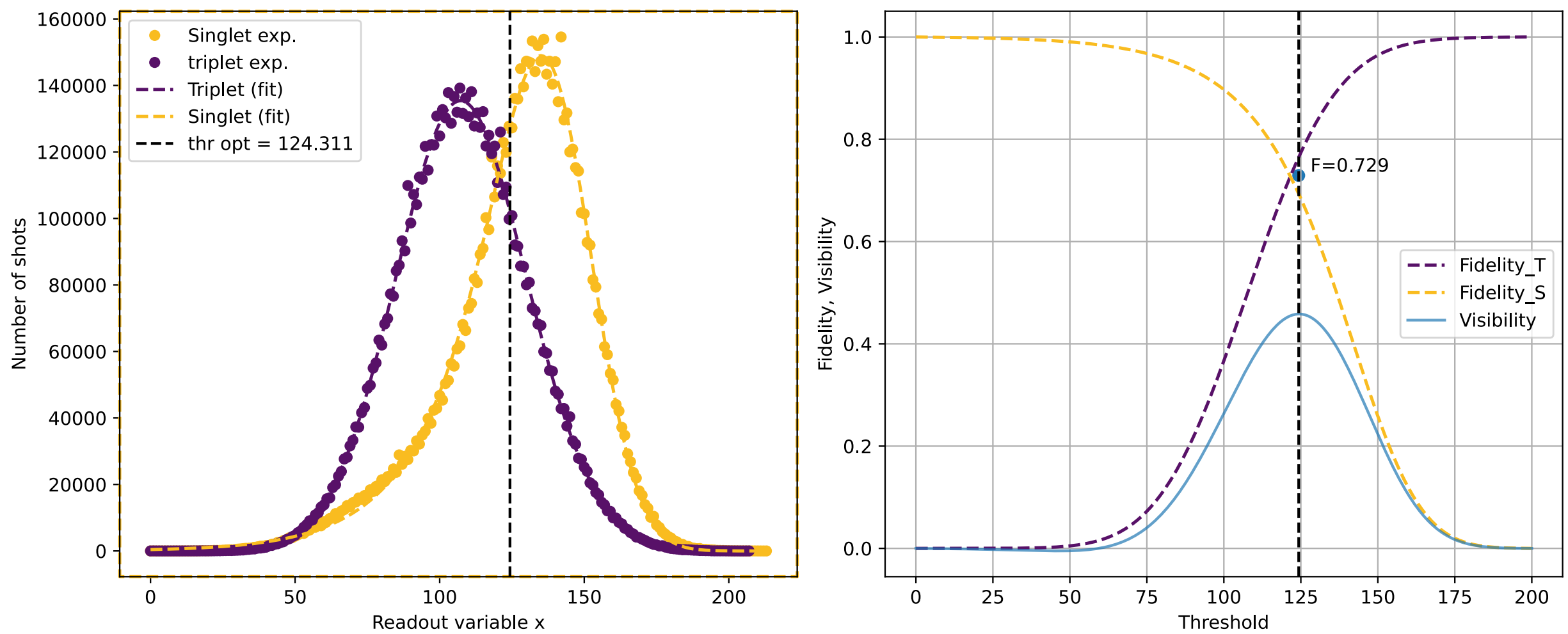

Supplementary Information Figure 3: Threshold analysis for the single shot readout at 1K The triplet and singlet distributions are fitted using a simple gaussian and a gaussian convoluted with an exponential decay respectively (see eq. 1 and 2). The threshold for state discrimination is set at the maximum of visibility. Here we obtain an average fidelity of 72.9(4)% for a visibility of 48.2(4)% in close agreement with the visibility extracted from the fitting of coherent oscillations.

which arises from the diagonalization of the toy-model Hamiltonian for the S(0,2), S(1,1) and T(1,1) states [2]. From the fit, we obtain $\tau_{2p} \approx 10\mu$eV. In the simulations, we biased the two face-to-face gates V_R, V_L to create a double QD, and we grounded all other gates in the device. In order to define a bias point in the simulations that is meaningful for comparison with the experiment, we chose the R and L gate bias to match the experimental $\tau_{2p} \approx 10\mu$eV. In Fig. 1(e), we estimate the two-particle tunnel coupling τ_{2p} as $\sqrt{2} * \tau_{1p}$, with τ_{1p} the tunnel coupling extracted from the single-particle calculation. This is reached for C=$(V_R + V_L)/2 = 0.674$ V, which is remarkably close to the experimental values shown in the main text (C = 0.8 V). We then scanned the exchange at the vicinities of the (1,1)-(2,0) anticrossing by performing individual FCI simulations. We plot the results, together with the experimental data, in Fig.7. There is a remarkable agreement between simulation and experiment, both in the magnitude of exchange and on the dependence on detuning. We attribute the slight mismatch in the values at small detuning to small differences in the experimental and simulated lever-arms. Note that the simulations are made in a pristine

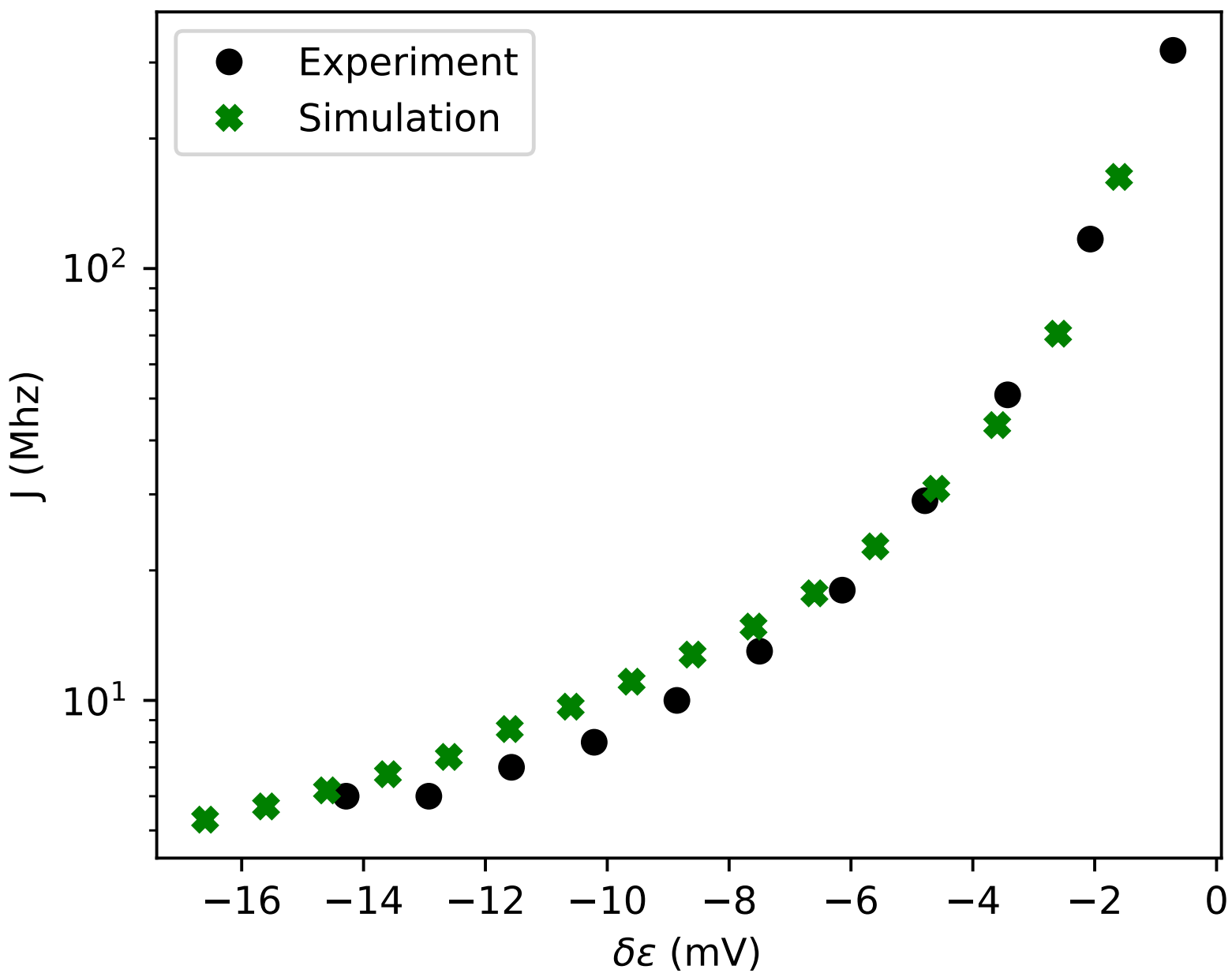

Supplementary Information Figure 4: Numerical simulation of exchange interaction Comparison between the simulated and experimental dependence of exchange on detuning. In the simulations, C=(V_R + V_L)/2 = 0.674 V.

device, in absence of electrical disorder. Several works have reported the strong impact of disorder on the QD properties [3, 4], as well as on the exchange interaction [5]. It is also important to point out that there are no fitting parameters in the simulation-experimental comparison. These results highlight once again the quality of the experimental device, whose electrostatics behave remarkably similar to the simulated one for a pristine device.

SUPPLEMENTARY INFORMATION SECTION 5 : EVOLUTION OF THE DISPERSIVE SIGNAL WITH TEMPERATURE

To model the temperature dependence of the dispersive shift, we compute the quantity χ within the standard dispersive approximation of a two-level system (here a charge qubit formed by an electron tunneling between two quantum dots) coupled to a harmonic resonator. The qubit–resonator interaction is described by the coupling rate g, and the

dispersive shift is given by

$$\chi = \frac{g^2}{\Delta} \tanh\left(\frac{\hbar\omega_q}{2k_\mathrm{B}T}\right), \tag{7}$$

where $\Delta = \omega_q - \omega_r$ is the detuning between the qubit frequency ω_q and the resonator frequency ω_r, and T denotes the electronic temperature. The hyperbolic tangent term accounts for the thermal occupation of the qubit and ensures that the dispersive shift vanishes at high temperature.

In the simulation, we sweep the tunnel coupling of our double quantum over a range of values (2–6 GHz) and the temperature between 100 mK and 1 K. For each pair of parameters, we compute χ on a two-dimensional grid and convert the result into frequency units. The quantity $\log_{10}(\chi)$ is then plotted as a color map as a function of the tunnel splitting and the temperature. This representation highlights how the dispersive response is degraded as temperature increases, in agreement with the experimental data. In particular for a tunnel coupling of 2.5 GHz, g=10 MHz and a resonator frequency of 1 GHz, we obtain a decrease of the dispersive shift of a factor 6 between 100 mK and 1 K.

SUPPLEMENTARY INFORMATION SECTION 6 : EVOLUTION OF THE HADAMARD GATE WITH THE DURATION OF AN IMPERFECT PULSE.

We detail here the experimental constraints that currently prevent the implementation of randomized benchmarking or gate-set tomography in our device.

Our fast-pulse control is generated using custom room-temperature electronics based on a Xilinx RFSoC ZCU-216 platform. In its present configuration, the architecture used for arbitrary waveform generation limits the complexity and flexibility of the pulse sequences that can be reliably synthesized. Although we are actively developing an improved control stack, the current combination of hardware and software does not permit the execution of the long, calibrated pulse sequences required for arbitrary gate sequences.

A central limitation arises from our inability to compensate the AC components of the control pulses. This is a consequence of the finite low-frequency cut-off of the bias tees used to combine DC and RF signals (100 kHz). The resulting high-pass filtering leads to a slow decay of the DC component of the pulses. Because the ratio between the difference of Zeeman energies between the two dots and the exchange energy is set by the position in detuning, this filtering has a significant impact on the Hadamard operation: the effective

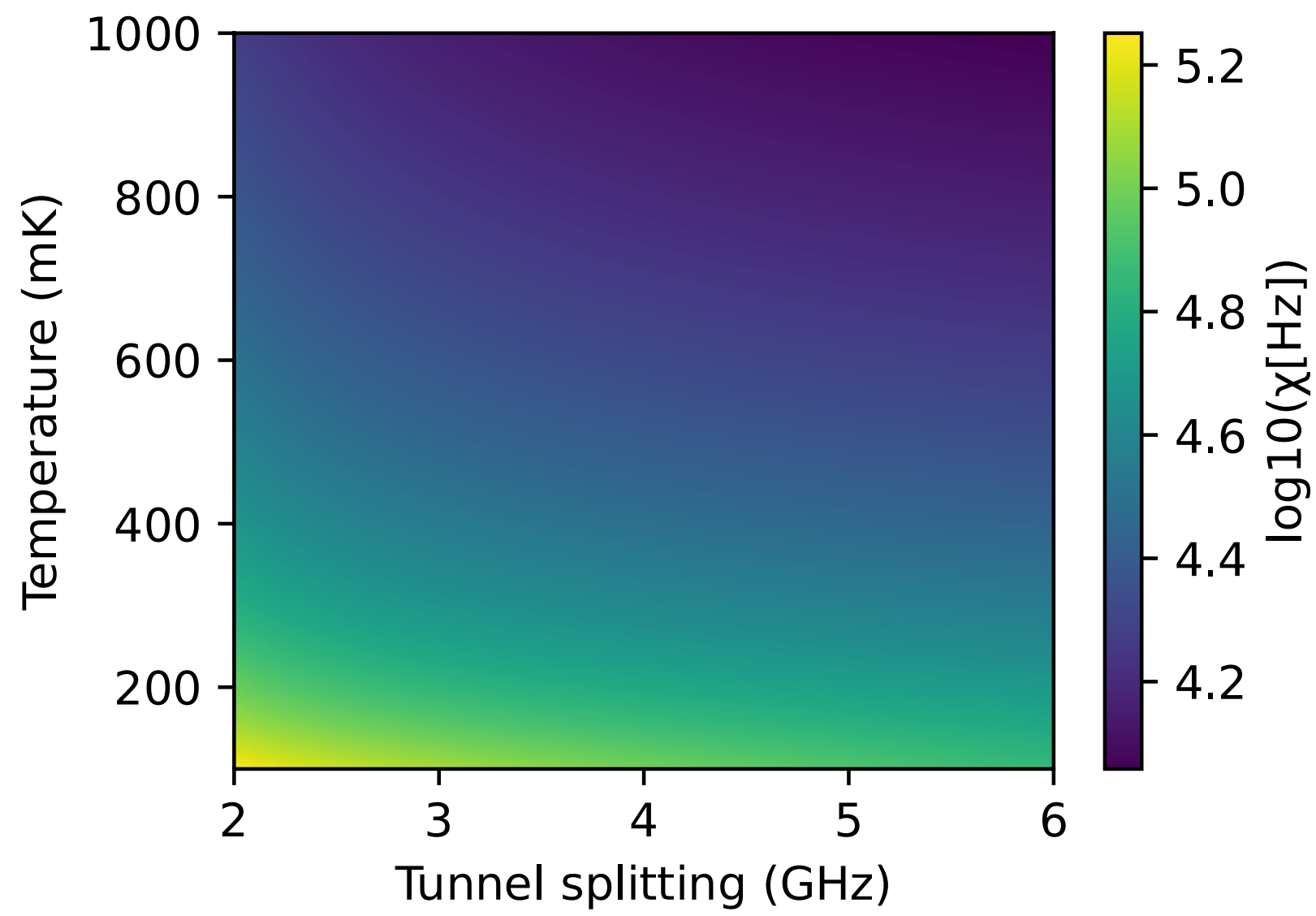

Supplementary Information Figure 5: Numerical simulation of the dispersive shift with temperature The color code represents the strength of the dispersive shift, χ, on the resonator calculated from 7. At equilibrium, the shift is proportional to the difference between the Boltzmann populations in the ground and excited singlet at zero detuning. The difference in energy between these two states is given by the tunnel splitting.

rotation axis is shifted away from the ideal $\pi/4$ axis in the YZ-plane of the Bloch sphere, see Fig. 5 (a). This effect is purely technical, and does not reflect any intrinsic limitation of the qubit itself. Using state-of-the- art arbitrary waveform generators with DC-coupled lines would eliminate this issue. To illustrate this point, we plot here the time evolution of the nominal Hadamard gate over extended durations and the corresponding simulation of the singlet population evolution. Both the simulation and the experimental traces reveal a reduction in oscillation visibility: the rotation axis drifts toward the north pole of the Bloch sphere, reducing visibility. Also we use a chirp-type of function to fit the experimental data to account for an increase of J as pulse amplitude decreases.

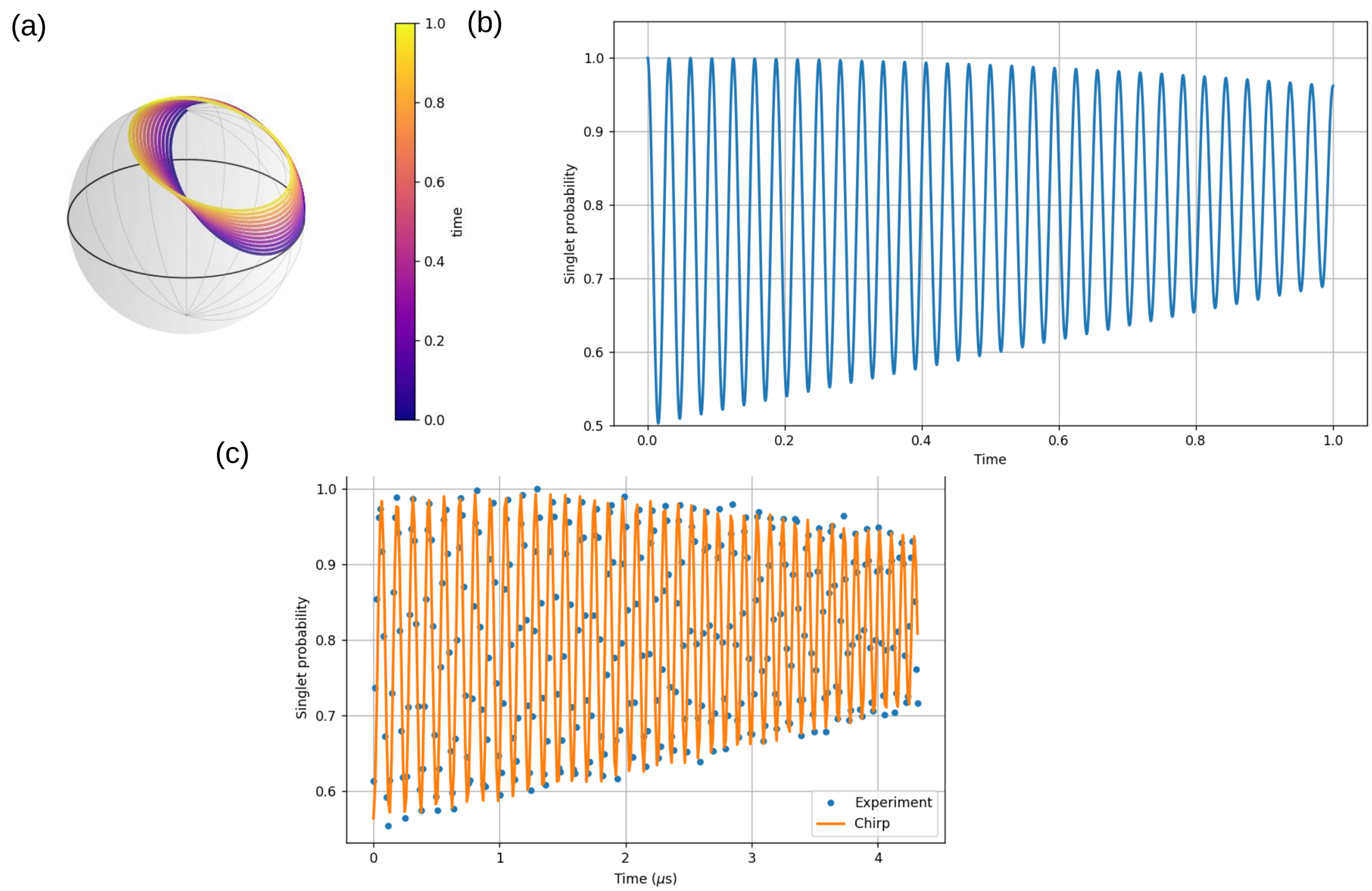

Supplementary Information Figure 6: Evolution of Hadamard gate with pulse duration. (a) Evolution of the rotation around an axis which starts at $\pi/4$ in the YZ plane and finishes at $\pi/10$. (b) Corresponding evolution of the oscillations visibility as a function of the pulse duration. (c) Experimental data (blue points) of an Hadamard like gate over 4μs duration. The initial detuning position corresponds to the point where the exchange energy matches the difference in Zeeman energy between the two dots. The fit in orange uses the same decay for the visibility decrease as the simulation and a linear dependence of the frequency oscillations is used to account for the variation in J with the pulse duration.

SUPPLEMENTARY INFORMATION SECTION 7 : EVOLUTION OF SNR WITH INTEGRATION TIME.

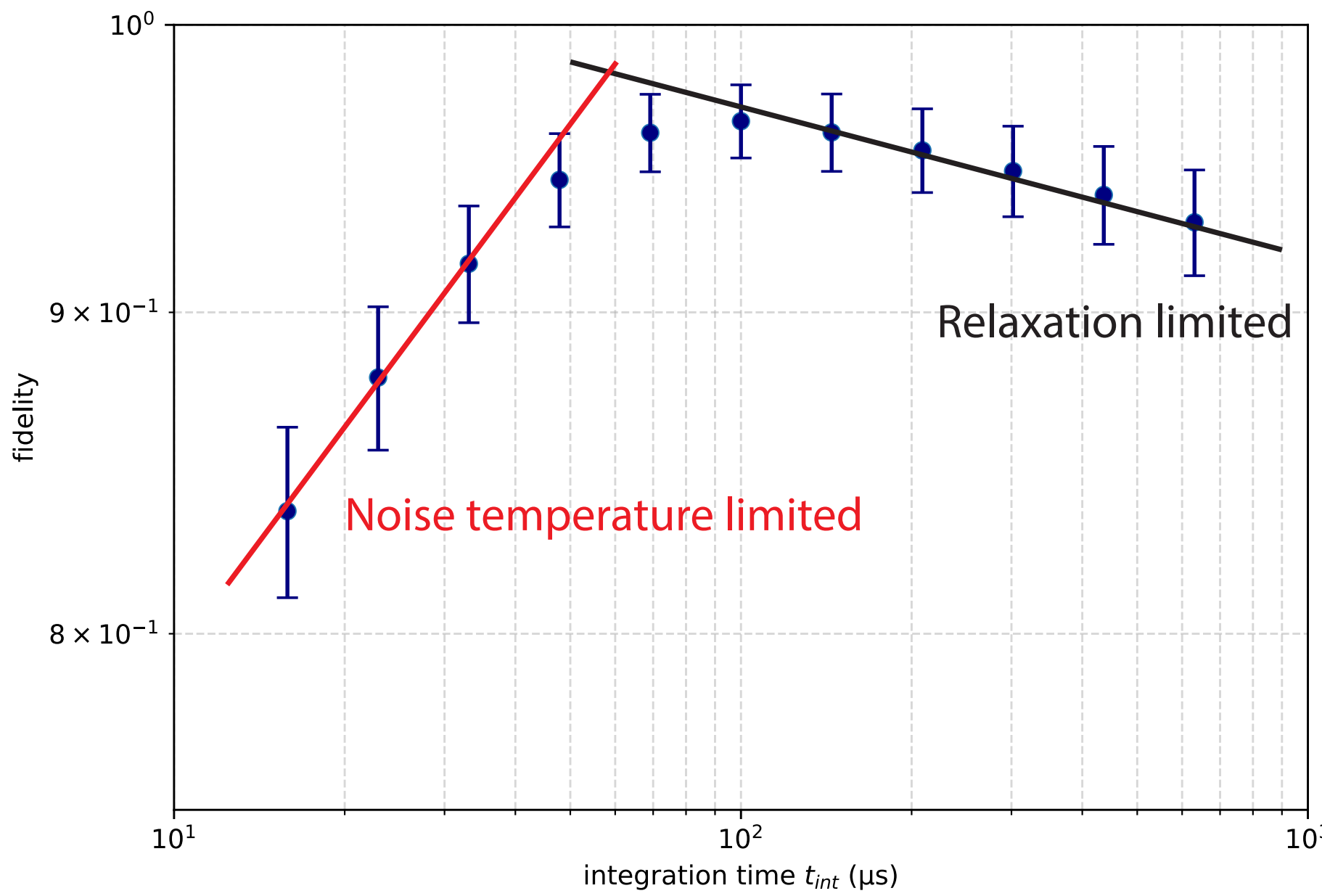

Supplementary Information Figure 7: Evolution of the fidelity as a function of the integration time. Average readout fidelity at the maximum of visibility is extracted as a function of the integration time. For short times, the infidelity is fit (red line) with a model governed by the noise temperature of the amplification chain while at long times it is fit (black line) with a model mostly dominated by relaxation effect during the measurement. In the present case, the optimal point is at 100μs. The error bars are extracted from the 5% interval confidence from the distribution fit. This fits include the Gaussian noise from the amplification chain and the decay induced by relaxation. The large error bar is mostly related to the relatively small amount of samples for these distributions (2000) and the difficulty to fit at small-fidelity-distributions.

SUPPLEMENTARY INFORMATION SECTION 8 : IMPACT OF THE MAGNETIC FIELD ON THE NIOBIUM INDUCTORS

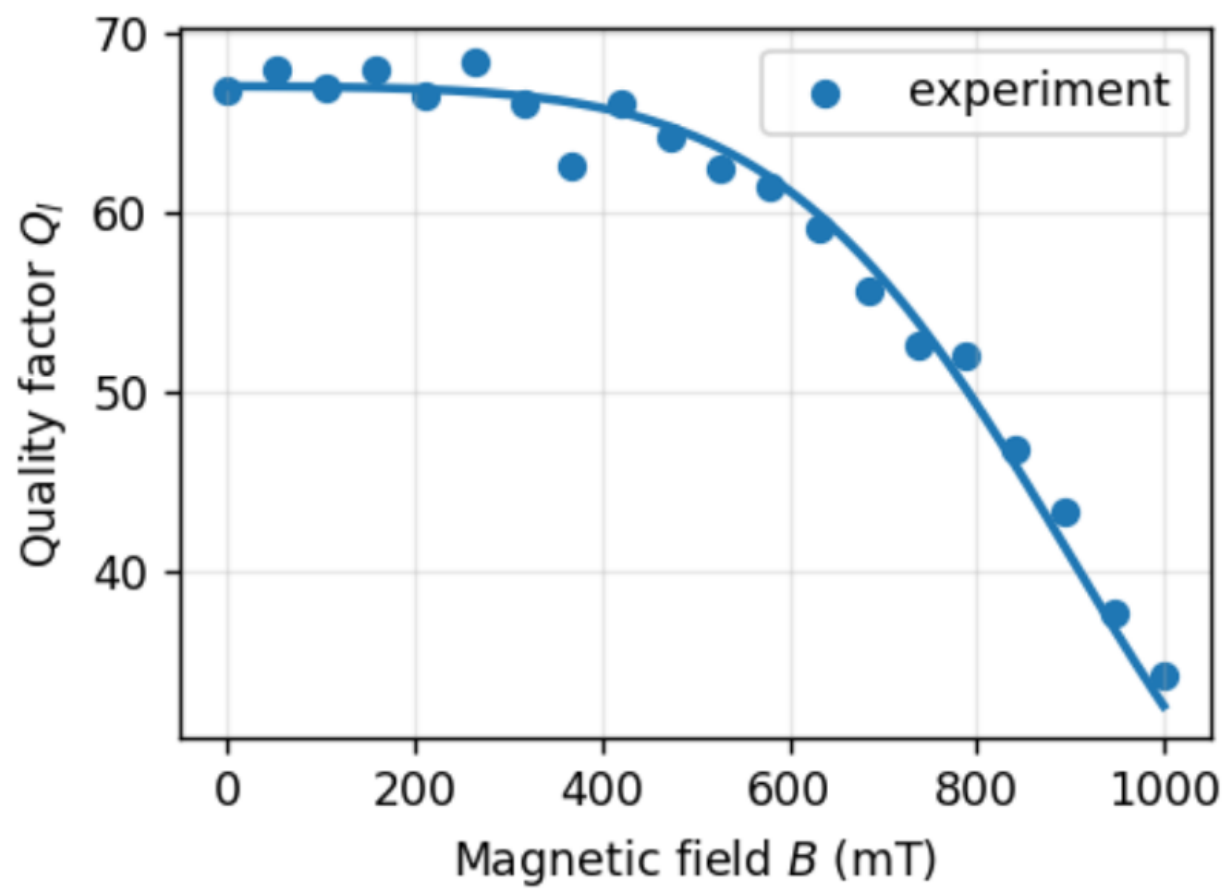

Supplementary Information Figure 8: Evolution of the quality factor of a resonator similar to the one used with respect to magnetic field. Dots correspond to the loaded quality factor extracted from experimental data, while the line serves as a guide to the eye. This resonator uses the same inductor as in the present experiment, but with a different qubit device providing the capacitance. Here, the resonance frequency is 856 MHz. The degradation of the quality factor is attributed to defects in the niobium layer that introduce additional losses. Efforts are currently underway to improve the quality of the deposited thin film, and mitigation strategies, such as reducing the resonator width, are also being investigated.

[1] C. Barthel, D. J. Reilly, C. M. Marcus, M. P. Hanson, and A. C. Gossard, Rapid single-shot measurement of a singlet-triplet qubit, Physical Review Letters **103**, 160503 (2009).

[2] G. Burkard, D. Loss, and D. P. DiVincenzo, "Coupled quantum dots as quantum gates," Phys. Rev. B **59**, 2070 (1999).

[3] B. Martinez and Y.-M. Niquet, Variability of electron and hole spin qubits due to interface roughness and charge traps, Phys. Rev. Appl. **17**, 024022 (2022).

[4] J. D. Cifuentes, T. Tanttu, W. Gilbert, J. Y. Huang, E. Vahapoglu, R. C. C. Leon, S. Serrano, D. Otter, D. Dunmore, P. Y. Mai, F. Schlattner, M. Feng, K. Itoh, N. Abrosimov, H.-J. Pohl, M. Thewalt, A. Laucht, C. H. Yang, C. C. Escott, W. H. Lim, F. E. Hudson, R. Rahman, A. S. Dzurak, and A. Saraiva, Bounds to electron spin qubit variability for scalable cmos architectures, Nature Communications **15**, 4299 (2024).

[5] B. Martinez, S. de Franceschi, and Y.-M. Niquet, Mitigating variability in epitaxial-heterostructure-based spin-qubit devices by optimizing gate layout, Phys. Rev. Appl. **22**, 024030 (2024)

.
